# Estradiol Receptors Regulate Differential Connexin 43 Expression in F98 and C6 Glioma Cell Lines

**DOI:** 10.1371/journal.pone.0150007

**Published:** 2016-02-26

**Authors:** Zahra Moinfar, Hannes Dambach, Bodo Schoenebeck, Eckart Förster, Nora Prochnow, Pedro Michael Faustmann

**Affiliations:** 1 Department of Neuroanatomy and Molecular Brain Research, Ruhr-Universität Bochum, 44801, Bochum, Germany; 2 International Graduate School of Neuroscience (IGSN), Ruhr-Universität Bochum, 44801, Bochum, Germany; Universidade Federal do ABC, BRAZIL

## Abstract

**Introduction:**

Glioma is the most common malignant primary brain tumour with male preponderance and poor prognosis. Glioma cells express variable amounts of connexin 43 (Cx43) and estrogen receptors (ERs). Both, Cx43 and ERs, play important roles in cell proliferation and migration. Therefore, we investigated the effects of 17-ß estradiol (E2) on Cx43 expression in two glioma cell lines with variable native expression of Cx43.

**Materials and Methods:**

F98 and C6 rat glioma cells were cultured for 24 h in the presence of 10 nM or 100 nM E2, and the E2-antagonist, Fulvestrant. An MTT assay was performed to evaluate cell viability. ERα, ERβ and Cx43 protein expressions were analysed by western blotting and Cx43 mRNA expression was analysed by real-time polymerase chain reaction. To quantify cell migration, an exclusive zone migration assay was used. Functional coupling of cells via gap junctions was examined using whole-cell patch-clamp technique.

**Results:**

E2 reduced Cx43 expression in C6 cells, but increased Cx43 expression in F98 cultures. These effects were mediated via ERs. Moreover, E2 promoted C6 cell migration, but it did not affect F98 cell migration. The expression level of ERα was found to be high in C6, but low in F98 cells. ERβ was exclusively expressed in C6 cells. In addition, E2 treatment induced a significant decrease of ERβ in C6 cultures, while it decreased ERα expression in F98 glioma cells.

**Discussion:**

These findings show that E2 differentially modulates Cx43 expression in F98 and C6 glioma cells, likely due to the differential expression of ERs in each of these cell lines. Our findings point to the molecular mechanisms that might contribute to the gender-specific differences in the malignancy of glioma and could have implications for therapeutic strategies against glioma.

## Introduction

Glioma is the most common primary malignant brain neoplasm [1]. Despite the low incidence of glioma, it is highly lethal with the five-year survival ranging from 4.7% in glioblastoma to 97% in pilocytic astrocytoma [2]. Epidemiological data show that glioma is up to two times more frequent in males than in females [1, 3, 4]. Experimental studies have shown an increased survival of male rats during early glioma tumour progression, once they were treated with estradiol [5]. Moreover, premenopausal women have longer survival than men, a difference that fades at postmenopausal stages [4]. These findings imply direct or indirect effects of sex hormones, namely female sex steroids, in glioma progression.

Connexin 43 (Cx43) is the most abundant gap junction (GJ) channel protein in astrocytes [6]. The GJ channels are formed by connecting connexons of adjacent cells, allowing a rapid exchange of molecules, such as mRNA or ions, through a network of GJ-connected cells. Since Cx43 is implicated in cell proliferation, migration and adhesion [7, 8], it has attracted attention as a therapeutic candidate molecule for glioma therapy.

Data on the influence of sex steroid hormones, specifically estradiol, in glioma cells are inconsistent. However, a variety of functions of steroid hormones have been proposed, ranging from preventive [9] to ineffective [10]. Estrogen, for example, can increase the survival of glioblastoma *in vivo* while ovariectomy abolishes this effect [5]. The mechanisms by which estrogen exerts its effects in glioma are still under investigation. Multiple functions of estradiol receptors (ERs), ERα and ERβ, for instance, have been suggested to mediate the various and often contradictory effects of estrogen on glioma [11, 12]. Moreover, Cx43 gene expression has been shown to be increased in estrogen-induced myometrium cells [13], while it was not altered in myocardial cells [14], suggesting a cell type-dependent Cx43 response to estrogen. The overexpression of Cx43 could have several opposing effects on tumour progression, ranging from a tumour suppressor gene function [15] to a modulatory role in cell migration and proliferation [7, 8]. Overexpression of Cx43, for example, is inversely correlated with the malignancy grade of glioma of astrocytic origin [16].

How Cx43 expression is influenced by estrogen in glioma cells remains an open question. Therefore, we investigated the regulatory effects of 17-ß Estradiol (E2) on two rat glioma cell lines. These cells were intentionally selected because they exhibit different native levels of Cx43 expression and GJ communication (GJC): C6 express low [17] and F98 high [18] levels of Cx43 expression, respectively. In addition, these cells mirror different categories of glioma: glioblastoma (F98) and astrocytoma (C6). Moreover, both cell lines are of rat origin, which facilitates the comparison of the results. Firstly, we evaluated the characteristics of ERs on both cell lines. Then, we analysed the effects of E2 on Cx43 expression by western blotting (WB) and Real-Time polymerase chain reaction (RT-PCR). Furthermore, we applied whole cell patch-clamp technique to study functional coupling under E2 treatment. We also used an exclusive zone migration assay to investigate the role of E2 on cell migration. Our findings imply a differential role for E2 on Cx43 modulation in a cell line-specific manner, which is, at least in part, due to a differential expression of ERα and ERβ in these cell lines.

## Materials and Methods

### F98 and C6 cell culture

F98 (CRL-2397™) and C6 (CRL-2199™) cells were provided from ATCC®. F98 cells were cultivated in minimum essential medium (MEM; Gibco) enriched with 10% foetal calf serum, 1% non-essential amino acids, penicillin (50 μg/ml) and streptomycin (50 μg/ml). C6 cell lines were cultured in Dulbecco’s minimal essential medium (DMEM; Gibco) with 1% glucose, 10% foetal calf serum, 1% non-essential amino acids, penicillin (50 μg/ml), streptomycin (50 μg/ml) and glutamine (2 mM, for C6 cells). Both cell types were kept continuously in stock culture dishes in 4–7% CO2, 93% air atmosphere at 37°C and nearly 100% humidity. The cells were regularly passaged twice a week and were used for a maximum period of four months. Subsequently, new aliquots were thawed and used upon need for further experiments. Medium was removed for this purpose and the cells were washed briefly with phosphate-buffered saline (PBS). The culture flasks were then treated with 0.1% EDTA for 5–10 min in the incubator and the detached cells were collected by adding new medium and centrifugation at 200 x g for 5 min. The medium was then removed and the cells were resuspended in new medium and transferred into appropriate flasks, culture dishes or multi-well plates until use.

### Western blotting

A total number of 40.000 cells/well (F98 or C6) were seeded in 3.5 cm culture dishes (BD bioscience). Four days after seeding, the cultures reached about 100% confluence and were treated with 10 nM or 100 nM E2 (Sigma) for 24 h; negative controls remained untreated. The E2 concentrations in these experiments were chosen based on previous studies on E2 effects, or being close to the E2 concentration during pregnancy in human plasma (a high, though physiological plasma concentration)[19–22]. Nexts, the wells were washed once with PBS and subsequently lysed and scraped from the dishes with 200 μl Laemmli 1 x buffer [(6.2%, (v/v) 0.5 M Tris–HCl pH 6.8; 2% (w/v) sodium dodecyl sulphfate (SDS); 10% (v/v) glycerol; 5% (v/v) b-mercaptoethanol plus)] and 4 μl protease inhibitor cocktail (Sigma). The lysates were kept on ice and measured for protein concentration by Bradford assay [23] (Bio-rad Bradford Protein Assay), according to the protocol, and 10 μg of the solution was loaded onto 10% SDS gel. The electrophoresis was performed for 20 min at 100 V, followed by 1 h at 150 V. The gels were transferred to nitrocellulose membrane at 90 mA/gel and remained for 1 h. Membranes were then blocked with Odyssey blocking buffer (LI-COR Bioscience) for 1 h and incubated with anti-β-actin (Sigma), anti-Cx43 (Sigma), anti-ERα or anti-ERβ (Santa Cruz) antibodies at 4°C overnight. The next day, they were washed with 0.1% Tween®20 (Applichem) in PBS (PBST) for 3×20 min and treated with secondary anti-mouse and anti-rabbit fluorescent antibodies (IRDYe 680 and 800, LI-COR Bioscience) for 1 h. Membranes were washed with PBST and the bands were visualised using an Odyssey Infrared Imaging System (LI-COR Bioscience), quantified with the ratio to beta-actin bands and normalized to the controls. Since fulvestrant (Sigma) is a potent competitive ER antagonist, it was used for some experiments at 1 μM, together with E2 for 24 h. Western blot analyses were performed according to the protocol described in section 0.

### MTT assay

The MTT assay is based on the cleavage of the yellow tetrazolium salt (3-[4,5-dimethylthiazol-2-yl]-2,5-diphenyltetrazolium bromide) to purple formazan crystals by metabolically active cells, which is measured with a microplate reader. According to MTT assay kit (Roche), the cells were incubated in 96-well plates, which had been coated previously with poly-L-lysine (PLL). At the fourth day, the cells were incubated with only carrier solutions, 10 nM or 100 nM E2 for 24 h, and then with 10 μl MTT reagent. After 4 h, the cells were incubated with 100 μl of solubilisation solution and kept overnight in the incubator. The next day, the viability of the cells in each well was measured by means of a microplate reader (Bio-rad) at 550 nm (A _550 nm_).

### RNA isolation from cell cultures

A total number of 40,000 F98 or C6 cells were seeded in 3.5 cm petri dishes and treated after four days with E2 for 24 h at 10 nM or 100 nM, or treated with only carrier solutions. The RNAs were isolated according to the protocol of the peqGOLD RNAPureTM System (Peqlab). Briefly, the cells were incubated with peqGOld reagent for 5 min at room temperature. Then, 0.2 ml chloroform was added, and the tubes were closed and vigorously shaken for 15 s. After 5 min at 4°C, they were centrifuged at 12,000 x g for 15 min at 4°C to obtain a three-phase solution. Next, the colourless upper aqueous phase of solution was separated and mixed with an equal volume of isopropanol for 15 min and centrifuged for 15 min at 12.000 x g (4°C). The RNA precipitate (a white-yellow pellet at the bottom of the tube) was isolated by removing the supernatant and washing once with 75% ethanol. The tubes were vortexed and centrifuged for 10 min at 12,000 x g (4°C). Finally, the excess of ethanol from the RNA pellet was removed by air-drying or by placing the sample under vacuum for 5–10 min. A high-yield RNA concentration was achieved with this protocol and the RNA concentration was measured using a spectrophotometer.

### Real time polymerase chain reaction (RT-PCR)

One-step RT-PCR was performed using a QuantiTect®SYBR®Green kit (Qiagen), according to the manufacturer’s protocol. Primers for *Cx43* and *GAPDH* (as an internal control) were purchased from Qiagen® Primer Assay. The final reaction volume of 50 μl per well was prepared by adding QuantiTect SYBR Green Master Mix (25 μl), Cx43 and GAPDH primers (each 5 μl), Quantitect RT Mix (0.5 μl), RNA template (5 ng) and a variable amount of RNase-free water (Gibco, Germany). The wells were subjected to a RT-PCR cycler (Opticon 2, Bio-rad) for 30 min at 50°C (for reverse transcription) and 15 min at 95°C for initial activation steps. Denaturation (15 s, 94°C), annealing (30 s, 50–60°C) and extension (30 s, 72°C) were repeated for 39 cycles. Data were extracted by setting the threshold at 0.017 and ΔCT was calculated based on the differences between the *Cx43* and *GAPDH* curves at this threshold.

### Functional coupling study

Forty thousand F98 or C6 cells were seeded on coverslips in 24-well plates. Four days after seeding, the medium was changed and the wells were incubated with 10 nM or 100 nM E2; controls were treated with only carrier solutions. The whole-cell patch-clamp technique (HEKA EPC 9 patch-clamp amplifier) was used to study Cx43 functional coupling. In order to evaluate the gap junctional intercellular communication (GJIC), patch pipettes (GB 150–8P; Science Products) with 5–10 MΩ resistance were filled with an intracellular solution [135 mM K gluconate, 20 mM KCl, 2 mM MgCl2, 10 mM 4-(2-hydroxyethyl)-1-piperazineethanesulfonic acid (HEPES), 10 mM ethylene glycol tetra acetic acid (EGTA); pH 7.2] containing 1% (w/v) Lucifer Yellow (LJ). Ringer solution was used as extracellular solution (B-Braun). The number of coupled glioma cells was counted 10 min after dye transfer from the injected cells to the neighbouring cells, using a Zeiss Axioskop equipped with a fluorescein isothiocyanate (FITC) filter set.

### Migration assay

An exclusive zone migration assay from OrisTM Migration Assay kit (Platypus) was used to study the migration of glioma cells under E2 influence. Briefly, 100.000 cells/well were seeded in 96-well plates in the presence of a stopper (2 mm diameter). The presence of the stopper prevented cell migration to the centre of the well. The stopper was removed after one day allowing the cells to migrate to the centre zone of the well. Upon stopper removal, the cells were incubated with 10 nM or 100 nM E2 for 24 h; controls were treated with only carrier solutions. On the third day, the medium was removed and replaced with phenol red-free medium containing Calcein (Invitrogen) for 30 min at 37°C. Cells were then visualised with an Axiovert microscope at 5 x magnification. The area covered with the cells in the centre zone (the precedent location of the stopper) was quantified by means of ImageJ software. A stopper in at least one well was maintained in its initial position in each set of experiments until the third day of incubation. The remaining stopper served as a reference to recognize the initial position in the wells where the stopper had been removed. The data were reported as the ratio of the area covered with cells to the area covered by the stopper.

### Transient transfection of C6 cells

Mouse Genomic DNA was used to amplify the coding region of Cx43 [24] by PCR by using the forward primer 5’-GCG GAA TTC ATG GGT GAC TGG AGC GCC TTG-3’ (EcoRI site underlined) and the reverse primer 5’-GCC GGA TCC AAT CTC CAG GTC ATC AGG CCG-3’ (BamHI site underlined). A PCR product of ~1145bp was separated on a 1.2% agarose gel and gel-purified using a Qiaex-II Gel extraction Kit (Qiagen). After digestion with EcoRI and BamHI (Fermentas), this fragment was ligated in-frame into the EcoRI/BamHI-digested pEGFPN3 vector to obtain a Cx43-eGFP fusion construct. After, C6 cells were transfected with Cx43-eGFP (green fluorescent protein) cDNA using the Effectene Transfection Reagent Kit (Qiagen). Cells were seeded on coverslips (12 mm ⌀) in 12-well plates at 40,000 cells/well. The next day, the cells reached about 70–80% confluence, and were transfected with 4 ng of Cx43-eGFP plasmid DNA. Using this protocol, about 70% of the cells were shown to express Cx43-DNA, as evaluated by fluorescence microscopy examination [24]. The medium in the wells was changed the next day and replaced with medium containing 10 nM or 100 nM E2. Controls were treated with only carrier solutions. After 24 h, the cells on the coverslips were lysed and subjected to western blot analysis, as described in section 0.

### Statistical analysis

Data were analysed with GraphPad Prism 5 (GraphPad Software, Inc, Version 5.04) and Origin Pro 9.1 (OriginLab Corporation) using One-way analysis of variance (One-way ANOVA) and Bonferroni post hoc test for parametric data and Kruskal-Wallis with Dunn’s post-hoc test for non-parametric data. Data are shown as mean ± SEM or mean ± SD and F with 95% confidence interval. The significance was set at 0.05.

## Results

### Differential expression of ERα and ERβ in F98 and C6 cells

**Fig 1:**
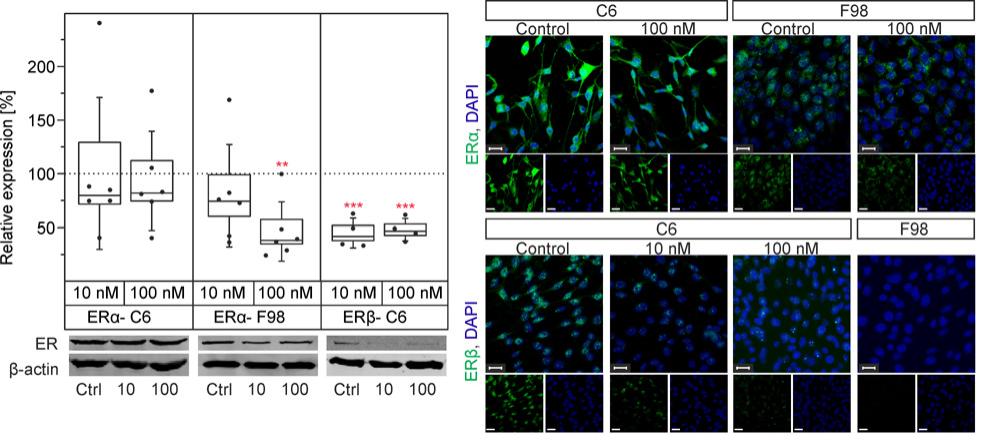
Western blot and immunocytochemistry of ERα and ERβ and their modulatory effects on F98 and C6 cells. Note the cytoplasmic expression of ERα in both cell lines. ERβ is detected in the nucleus of C6 cells. Ctrl (Control); 10 (10 nM E2); 100 (100 nM E2); **: p ≤ 0.01, ***: p ≤ 0.001. Boxes represent SEM and median, whiskers represent ± SD. The number of experiments is represented by dots over boxes. Scale bars: 20 μm.

Both cell lines expressed ERα, as assessed by WB. However, C6 cells expressed ERα more prominently than F98 cells (Fig 1). Moreover, C6 cells weakly expressed ERβ, while F98 cells did not express this receptor. ERβ expression significantly decreased (p < 0.01, n = 6) in C6 cultures treated with 10 nM (44.9 ± 6.7%) or 100 nM E2 (48 ± 5.1%) compared to controls (100%). There was no significant change of ERα expression in C6 cells under treatment with E2, although ERα expression significantly decreased (p < 0.01, n = 6) after treatment with 100 nM E2 (46.2 ± 11.2%) compared to controls (100%).

### Differential regulation of Cx43 protein and mRNA expression in F98 and C6 cells

**Fig 2:**
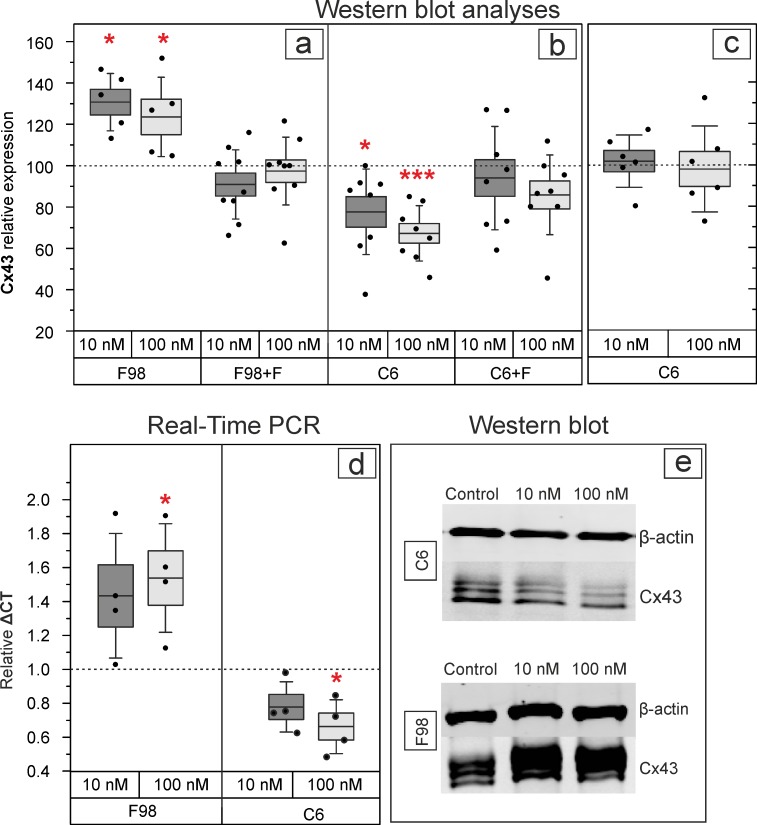
Relative Cx43 expression in C6 and F98 cells detected by western blot and RT- PCR analyses. a) F98 cells showed a significant increase and b) C6 cells showed a significant decrease of Cx43 protein expression after treatment with 10 nM or 100 nM E2. c) Transfection of C6 cells with Cx43-cDNA neutralized these effects. d) RT-PCR showed an opposite effect of E2 on Cx43 gene expression in C6 when compared to F98 cells. e) A representative WB showing the increased Cx43 protein expression in F98 cells and the decreased Cx43 expression in C6 cells, after E2 treatment. The number of experiments is depicted by the dots in the box plot. F: fulvestrant, *: p < 0.05, **: p ≤ 0.01, ***: p ≤ 0.001. Bars and whiskers represent mean ± SD.

**Fig 3:**
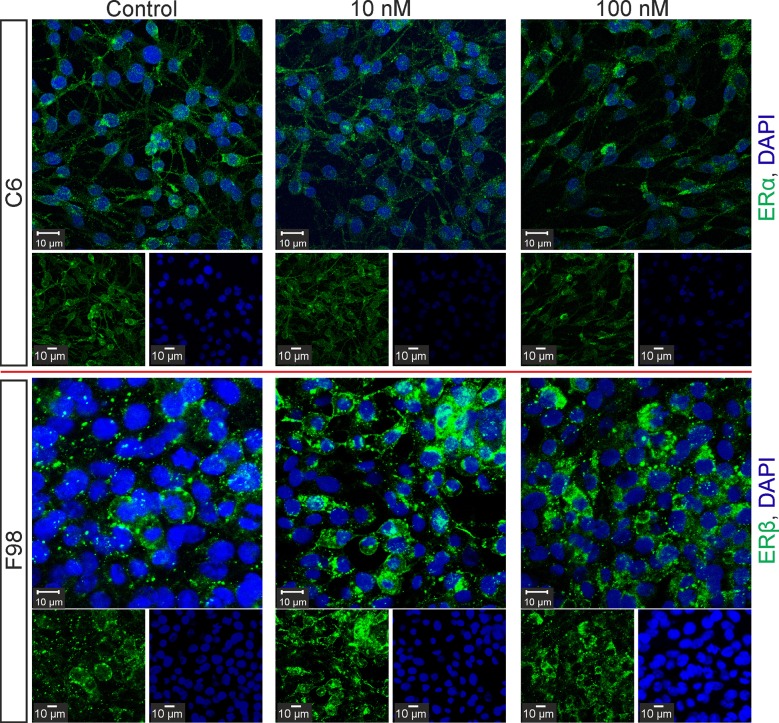
Immunocytochemical analyses of Cx43 protein expression in F98 and C6 cells. Note the decreased expression of Cx43 in C6 cells (upper panel) compared to the increased expression of Cx43 in F98 cells (lower panel), after E2 treatment. Both, cytoplasmic and membrane distribution of Cx43 is detectable in either cell lines after E2 treatment.

The WB analyses showed a significant increase of Cx43 expression in F98 cultures and a significant decrease of Cx43 expression in C6 cultures after treatment with E2 at 10 nM or 100 nM E2 in comparison to controls (Fig 2A and 2B, Fig 3 and Table 1). In addition, fulvestrant abolished these effects in both cell lines (Fig 2A and 2B). Similarly, RT-PCR revealed reduced Cx43 mRNA expression in C6 cells after treatment with 100 nM E2 (p < 0.05, ΔCT = 0.6 ± 0.07, n = 4), but increased Cx43 mRNA expression in F98 cells (p < 0.05, ΔCT = 1.5 ± 0.1, n = 4) after the same treatment in comparison to controls (Fig 2D). Moreover, E2 restored the decreased Cx43 mRNA expression level in C6 cells after transfection with Cx43-cDNA (Fig 2C).

**Table 1:** Results of statistical analyses of western blot, RT-PCR and cell migration assays.^1^

		**RT-PCR**		
		*Mean*	*SEM*	*p value*
**F98**	10 nM	1.4	0.2	ns
	100 nM	1.5	0.2	*
**C6**	10 nM	0.8	0.07	ns
	100 nM	0.7	0.07	*
		**Western Blot**		
		*Mean*	*SEM*	*p value*
**F98**	10 nM	130.5	8	*
	100 nM	123.3	11.1	*
**C6**	10 nM	77.6	7.4	**
	100 nM	67.1	4.8	***
		**Migration assay**		
		*Mean*	*SEM*	*p value*
**F98 (24 h)**	Control	7.2	0.4	Ns
	10 nM	9.1	0.9	Ns
	100 nM	8.2	0.8	Ns
**C6 (24 h)**	Control	12.7	0.9	ns
	10 nM	21.3	1.2	***
	100 nM	19.2	1.5	**
**F98 (48 h)**	Control	11.6	0.8	Ns
	10 nM	12	0.5	Ns
	100 nM	13.3	1.5	Ns
**C6 (48 h)**	Control	23	1.2	ns
	10 nM	30.1	1.9	*
	100 nM	31.1	1.8	**

^1^All experiments were statistically tested by One-way ANOVA in parametric data followed by Bonferroni post-hoc test. For non-parametric data Kruskal-Wallis was used to test the significance followed by Dunn’s post-hoc test within groups. RT-PCR and western blot controls are set as 1 and 100, respectively.

*: p < 0.05

**: p ≤ 0.01

***: p ≤ 0.001

ns: not significant.

### Differential effect of E2 on C6 and F98 cells in whole-cell patch-clamp studies

**Fig 4:**
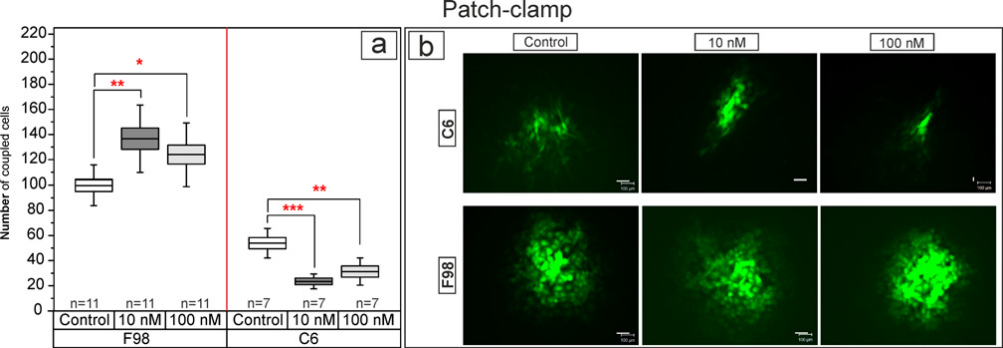
Differential effects of E2 on the functional coupling of C6 and F98 cells. a) Significant decrease of coupling of C6 cells and b) Significant increase of coupling of F98 cells after E2 treatment. Bars and whiskers represent mean ± SD, *: p < 0.05, **: p ≤ 0.01, ***: p ≤ 0.001. b) Photomicrographs show the number of coupled cells in F98 and C6 glioma cultures. The extent of coupling is reflected by the number of luminescent cells.

The coupling of cells via gap junctions in C6 and F98 was oppositely regulated by E2 (Fig 4). The absolute number of coupled F98 cells (100 ± 16, mean ± SD) significantly increased (p = 0.002, F = 7.5, n = 11) to 137 ± 26 when treated with 10 nM and to 124 ± 25 cells when treated with 100 nM E2. By contrast, E2 significantly decreased (p = 0.0002, F = 15, n = 7) the number of coupled cells in C6 cells from 54 ± 11 in controls to 24 ± 6 when treated with 10 nM and to 31 ± 10 when treated with 100 nM E2.

### 17- ß estradiol promoted cell migration in C6 but not in F98 cells

**Fig 5:**
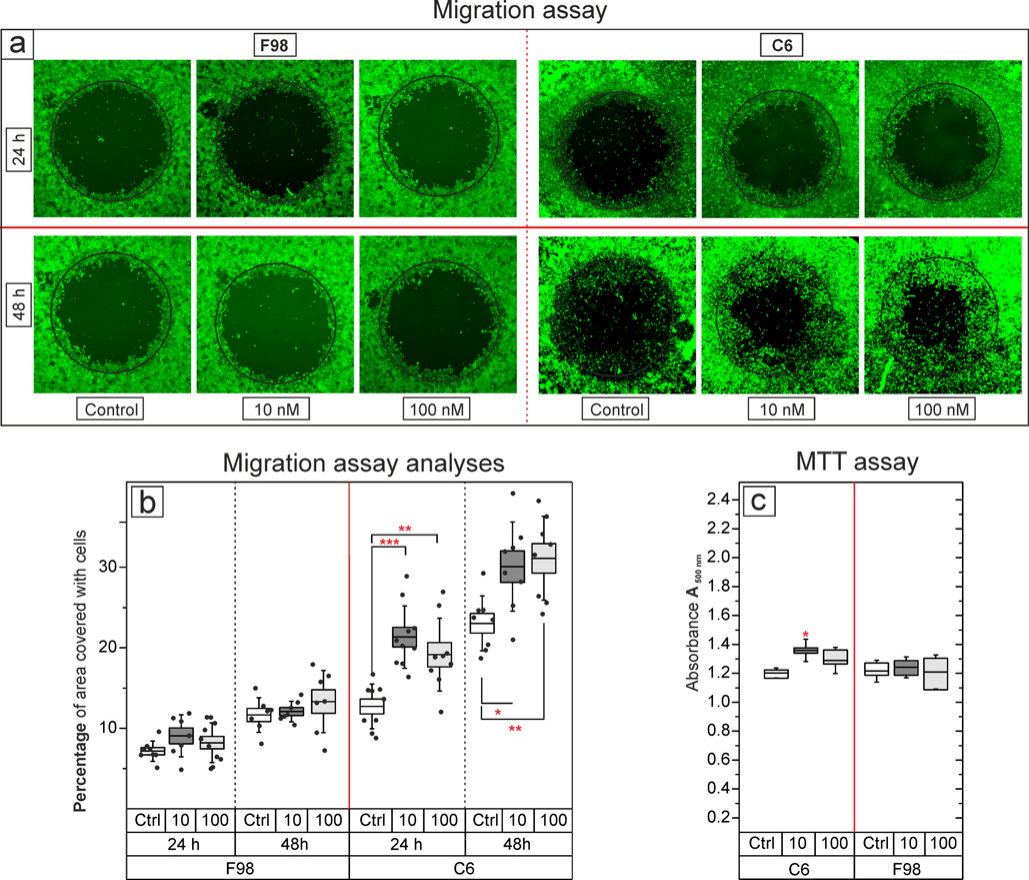
Exclusive zone migration assay and MTT assay in C6 and F98 cultures after E2 treatment. a) Photomicrographs of the exclusive zone migration assay in F98 and C6 cells after E2 treatment. The black line surrounding the dark circle in the middle depicts the location of the stopper prior to removal. b) Significant increase of C6 cell migration after incubation with 10 nM or 100 nM E2. Number of the experiments are represented by black dots over boxes. c) MTT assay shows increased C6 cell proliferation after treatment with 10 nM E2; n = 6–8, bars and whiskers represent mean ± SD, *: p < 0.05, **: p ≤ 0.01, ***: p ≤ 0.001. Ctrl: Control, 10: 10 nM; 100: 100 nM.

Migration of F98 cells did not significantly change after 24 h treatment with E2; however, migration of C6 cells was promoted (p = 0.0001, F = 13, see Fig 5A and 5B) when treated with 10 nM E2 (21 ± 4%, n = 10) or 100 nM E2 (19 ± 4%, n = 9) in comparison to controls (13 ± 3%, n = 9). Similarly, E2 promoted migration of C6 cells (p = 0.005, F = 6, n = 8) when treated for 48 h with 10 nM E2 (30 ± 5%) or 100 nM E2 (31 ± 5%) compared to controls (23 ± 3.4%).

### Increased cell proliferation in C6 cells in the MTT assay

**Fig 6:**
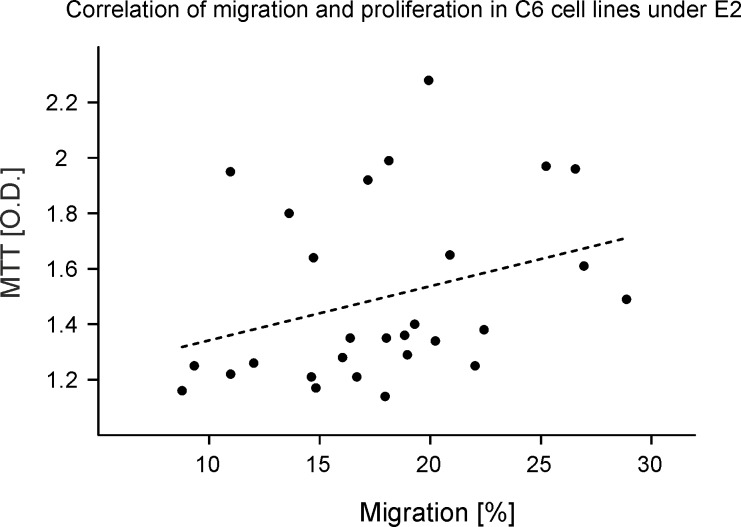
Correlation of migration and proliferation in C6 cell cultures after E2 treatment. Migration was evaluated according to the data obtained from the exclusive zone migration assay. The intermittent line shows the equation as follow: y = 0.02x + 1.1, R2 = 0.1.

The MTT assay showed differential regulation of cell proliferation in F98 and C6 cultures (Fig 5C). Cell viability was increased in C6 cultures (p = 0.006, F = 7.2) at 10 nM (1.4 ± 0.07) compared to controls (1.2 ± 0.03). However, cell viability was not significantly altered in F98 cultures (Fig 5C). Based on the technique that we used in this study (refer to section 0), an increased O.D. implies more viable cells and consequently the existence of more cells in the cultures. Correlation analysis was performed between these two parameters to exclude the effect of cell proliferation on migration of C6 cells. There was no significant correlation between cell migration and proliferation (p = 0.3, R2 = 0.1, see Fig 6).

## Discussion

**Fig 7:**
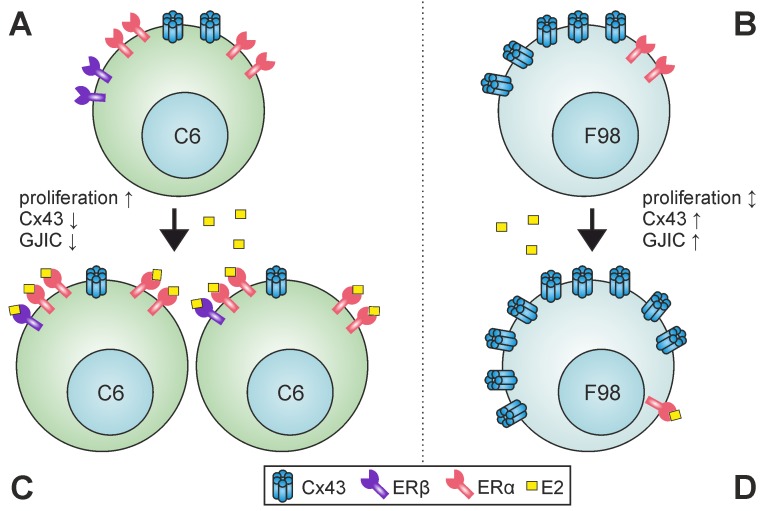
Schematic view of the differential effects of E2 on Cx43 expression in C6 and F98 cells. C6 and F98 cells respond oppositely to E2, an effect that is likely, in part, owed to the differential expression of ERs in each of these cell lines. While C6 cells express more ERα and less ERβ (A), F98 cells express only a weak amount of ERα but do not express ERβ (B). C6 cells down-regulate Cx43 (C) and F98 cells up-regulate Cx43 in response to E2 (D). GJIC: gap junctional intercellular communication, ↕: no significant change was observed.

Based on the suggested role of estrogen and Cx43 in glioma progression, the present study addressed the question of how estrogen influences Cx43 expression in two distinct glioma cell lines. Since GJs are implicated in cell proliferation and development [25, 26], understanding the role of GJs in cancer could be helpful to find new anti-neoplastic therapeutic agents. Findings related to GJ in glioma studies are often controversial, ranging from pro-/anti-proliferative to pro-/anti-apoptotic [27]. On the other hand, epidemiological data show that glioma is less frequent in females than in men [2], implicating a beneficial role of estrogen in glioma inhibition [5]. Although the effect of E2 on Cx43 has been specified previously [19], this effect has not yet been studied in glioma cells. Our findings show that E2 differentially regulates Cx43 expression, GJC, migration and proliferation of F98 and C6 rat glioma cell lines, and suggest a role of E2 for differential ERs expression in these cell lines (Fig 7).

Several lines of evidence support the inverse association of Cx43 expression, cell migration [7, 28, 29] and proliferation [16, 29, 30]. Consequently, our data fit the concept of “grow to go” in C6 cells [7], which supports the idea that enhanced migration and reduced Cx43 expression are accompanied by decreased cell coupling. However, F98 cells did not show reduced migration significantly, although Cx43 expression and the number of coupled cells were increased. The difference between F98 and C6 cell migration indicates that manipulation of Cx43 solely is not sufficient to modulate cell migration, and that, Cx43 modulation is influenced by the type of ERs that each individual cell line, such as F98 or C6, expresses.

Although a previous study showed the expression of ERβ but not ERα in C6 cells [31], our data provide evidence for the expression of both receptors in C6 cells, ERα, and, to a lower extent, ERβ. On the other hand, only a weak expression of ERα, and no expression of ERβ were found in F98 cells (Fig 1). Accumulating evidence suggests opposing roles of ERα and ERβ with regard to cell proliferation [32, 33]. Because of individual conformational changes of ERs upon ligand binding, ERα and ERβ may transduce opposing signals which are often proliferative in cells that express ERα, and apoptotic in ERβ expressing cells [11, 34, 35]. Similar findings propose that the ratio of ERα and ERβ expression, in a cell that co-expresses both ERs, might also code both anti-and pro-apoptotic signals in a tissue and cell type-specific manner [34]. Consequently, increased proliferation and migration of C6 cells accompanied by down-expression of Cx43 and decreased GJIC are compatible with the assumption that ERα enhances tumour growth. Moreover, ERβ was decreased in the same cell line, suggesting an inhibitory effect of ERβ on cell migration. Interestingly, ERα was decreased in F98 cells that do not express ERβ, but E2 neither modulated cell migration nor affected F98 proliferation. Although current findings point to the importance of the ER subtype, it seems more likely that the ratio of ERs expressed by individual cells governs their proliferation and migration, as well as the response to Cx43 expression under E2 [11, 34].

E2 affected GJIC in C6 cells oppositely when compared to F98 cells. The mechanisms that underlie these alterations include changes in Cx43 expression or changes in the phosphorylation state of existing channels. As inferred from the present study, modifications of Cx43 might, in part, be reflecting the type of ERs expressed by each cell line, resulting in the different responses of F98 and C6 cells. Here, we could show that these Cx43 modulations are mainly initiated at the genomic level, causing substantial changes in the number of channels and, thus, GJIC alterations in both cell types. However, alterations in channel phosphorylation, the essential step of channel permeability, are a subject that needs to be considered as well. In general, GJIC modulations might influence the exchange of specific mRNAs or ions, which due to their fast transfer within a cell network, may also inhibit certain growth characteristics of glioma tissue.

The findings of this study might help to unravel some aspects of glioma development in a gender-based manner. These data imply that the outcome of therapeutic approaches, such as those suicidal gene-therapies that benefit from Cx43 expression, might be gender-dependent. Moreover, E2 can influence the expression of growth factors in various ways through its modulatory effects on the regulation of a variety of genes [36, 37] such as differential modulations of growth factors or cytokine release in glioma cells (S1 Table).

Finally, we suggest a double-edged sword role for estrogen with regard to Cx43 in glioma pathology. The low expression of Cx43 in C6 cells combined with a tendency of these cells to migrate fits several glioma models. These findings might have implications in our understanding of glioma progression and might provide new therapeutic tools in the future. It also underscores the differential roles of E2 and its physiological consequences in glioma growth. The differential expression of ERs in F98 and C6 cells, which potentially induced opposing Cx43 modulations in response to E2, represent a new model to study gender-specific differences in glioma therapy. However, it should be kept in mind that these cell lines belong to different categories of glioma with major pathological differences. Accordingly, the discrepancies between the ER expressions of different glioma cells might be one of the causes, among other representative features, that underlie the differential modulation of Cx43 expression in these cells. These molecular characteristics of different glioma cell lines should not only be studied in more detail, but also in cultures where glioma cells are combined with other cell types, such as neurons, astrocytes or microglia.

## Supporting Information

S1 Table TGFβ and TNFα modulations in F98 and C6 cells under E2. TGFβ: Transforming Growth Factor beta; TNFα: Tumour Necrosis Factor alpha; TGFβ was not significantly modulated but it showed a tendency to increase in F98 and decrease in C6 cells. TNFα was not significantly modulated in any of the cell lines.
(DOCX)
