# Supplementary material for: Estradiol Receptors Regulate Differential Connexin 43 Expression in F98 and C6 Glioma Cell Lines
Source: PLoS One. 2016 Feb 26;11(2):e0150007. doi: 10.1371/journal.pone.0150007 (PMC4769248; doi:10.1371/journal.pone.0150007)
Supplement: S1 Table — TGFβ: Transforming Growth Factor beta; TNFα: Tumour Necrosis Factor alpha; TGFβ was not significantly modulated but it showed a tendency to increase in F98 and decrease in C6 cells. TNFα was not significantly modulated in any of the cell lines. (DOCX) [file pone.0150007.s001.docx]

Supplementary data. TGFβ^[[1]](#footnote-1)^ and TNFα^[[2]](#footnote-2)^ modulations in F98 and C6 cells under E2.

|  |  |  | **TGF-β^[[3]](#footnote-3)^** | | **TNF-α^[[4]](#footnote-4)^** | |
| --- | --- | --- | --- | --- | --- | --- |
|  |  | n | Mean  (pg/ml) | SEM | Mean  (pg/ml) | SEM |
| **F98** | Control | 7 | 1837.4 | 150.4 | 12.2 | 4.5 |
|  | E2 10 | 7 | 1616.2 | 118.1 | 17.0 | 6.8 |
|  | E2 100 | 7 | 1633.0 | 110.9 | 11.2 | 3.1 |
| **C6** | Control | 5 | 1284.2 | 68.1 | 12.5 | 4.9 |
|  | E2 10 | 5 | 1398.1 | 86.2 | 11.1 | 4.7 |
|  | E2 100 | 5 | 1384.4 | 86.6 | 8.6 | 4.8 |

1. Transforming Growth Factor beta [↑](#footnote-ref-1)
2. Tumor Necrosis Factor alpha [↑](#footnote-ref-2)
3. TGFβ was not significantly modulated but it showed a tendency to increase in F98 and decrease in C6 cells. [↑](#footnote-ref-3)
4. TNFα was not significantly modulated in any of the cell lines. [↑](#footnote-ref-4)
